# Faecal microbiota transplantation and glucolipid metabolic disorders: the interventional role of gut microbiota

**DOI:** 10.3389/fendo.2026.1806638

**Published:** 2026-04-16

**Authors:** Jiali Wu, Yusheng Qiu, Jiaying Deng, Yangpeng Li, Biao Jia, Zheng Cao, Jiaqing Tao, Jiao Guo

**Affiliations:** Guangdong Metabolic Diseases Research Centre of Integrated Chinese and Western Medicine, Key Laboratory of Glucolipid Metabolic Disorder, Ministry of Education; Guangdong Traditional Chinese Medicine Key Laboratory for Metabolic Diseases, Guangzhou Higher Education Mega Centre, Institute of Chinese Medicine, Guangdong Pharmaceutical University, Guangzhou, China

**Keywords:** faecal microbiota transplantation, glucolipid metabolic disorders (GLMD), gut microbiota, intestinal barrier, metabolic syndrome

## Abstract

Glucolipid metabolic disorders (GLMD) have emerged as a significant global public health issue, posing a significant threat to human health. With changes in modern social structures and an ageing population, the incidence of GLMD is on the rise and is increasingly affecting younger populations. Faecal microbiota transplantation (FMT) directly modifies the gut microbiota to reestablish its equilibrium and metabolites, consequently reinstating gut barrier integrity, mitigating chronic low-grade inflammation, and affecting the onset and progression of GLMD through the regulation of the gut-liver axis. This paper reviews the application of FMT in the treatment of GLMD, emphasizing research outcomes and efficacy assessments in clinical trials and animal studies. As a simple and secure intervention, FMT is anticipated to provide new therapeutic alternatives for GLMD patients in the future with the deepening of relevant research, the screening of specific probiotics and the revelation of functional mechanisms. This paper aims to clarify the potential mechanism of FMT in addressing GLMD, summarise recent research developments in this field, and anticipate the opportunities and challenges of FMT in clinical application.

## 1 Introduction

Glucolipid metabolic disorders (GLMD) is a category of diseases defined by abnormalities in glucose and lipid metabolism, which are affected by a confluence of genetic, environmental, psychological, and dietary factors. The fundamental pathogenesis includes neuroendocrine dysfunction, insulin resistance, oxidative stress, chronic inflammation, and intestinal flora dysregulation (1). Clinical symptoms encompass hyperglycaemia, dyslipidaemia, metabolic-associated fatty liver disease (MAFLD), obesity, hypertension, and atherosclerosis, frequently occurring alone or in conjunction. The rising incidence of these diseases annually presents a substantial threat to human health.

Studies have shown that the combination of diabetes, dyslipidaemia and non-alcoholic fatty liver disease not only increases the risk of microvascular complications and macrovascular complications, but also aggravates the complexity of the disease. A comprehensive treatment for glucolipid metabolic disorders remains difficult to achieve with current approaches. This is largely due to their reliance on single-disease strategies, which are ill-suited for managing common comorbidities including abnormal glucose metabolism, abnormal lipid metabolism, hypertension, and non-alcoholic fatty liver disease. As a result, how to coordinate treatment at the holistic level, particularly in identifying effective intervention points in the many pathogenic pathways of glucolipid metabolic diseases, has emerged as an important problem in contemporary medical study.

Recent studies have identified a significant correlation between intestinal flora dysregulation and the onset and progression of GLMD. The alteration of gut microbiota promotes lipid metabolism disorders and abnormal glucose metabolism by increasing the permeability of the intestinal barrier, inducing chronic low-grade inflammation and insulin resistance. Consequently, managing the equilibrium of intestinal microbiota is regarded as a new approach for the prevention and management of glucolipid metabolic diseases.

Faecal Microbiota Transplantation (FMT) is a therapeutic method that involves transferring the faecal microbiota from a healthy donor into a patient’s gastrointestinal tract via specific routes to treat diseases associated with dysbiosis of the gut microbiota (2–4). FMT has attracted increasing attention as a treatment method to reconstruct the balance of the recipient’s intestinal microbiota by transplanting the faecal microbiota of healthy donors. FMT not only improves intestinal barrier function and modulates immune responses, but has also been shown to have significant therapeutic effects in a variety of diseases. In recent years, more and more studies have explored the potential of FMT in the treatment of GLMD, showing its unique advantages in improving glucose metabolism, lipid metabolism and reducing chronic inflammation. This article aims to review the application progress of FMT in the treatment of GLMD, elucidating its specific mechanisms, such as the restoration of gut barrier integrity and the modulation of the gut-liver axis. In addition, we aim to evaluate the clinical effects and safety profiles observed in recent trials, and critically anticipate the future research directions and clinical challenges. Through this review, we hope to provide clear guidance for the future screening of specific probiotics and the optimization of FMT methodologies.

## 2 Gut-liver axis

The liver-gut axis serves as a bridge between the gut and the liver (5, 6). Under normal physiological conditions, nutrients, toxins and microbiota metabolites in the intestine enter the liver through the portal vein system, where they are metabolized and affect liver function. Bile acids and immunoglobulin A and other substances produced by the liver are excreted through the biliary system and enter the intestinal lumen to maintain the intestinal ecological balance.

Intestinal epithelial cells (IECs) are linked by dynamic tight junctions (TJs). TJs are a category of proteins predominantly consisting of claudins, occludin, zonula occludens-1 protein, and junctional adhesion molecules (JAMs) (7–12). TJs play a crucial role in maintaining the integrity of the intestinal epithelial barrier. TJs restrict the entry of pathogens by allowing the secretion of immunoglobulin A (IgA), thereby preventing IECs from being damaged by microbial pathogens; on the other hand, they allow various nutrients to enter the intestinal wall (13, 14). Additionally, the immune system plays a significant role, with the release of interleukin-23 (IL-23) inducing the activation of group 3 innate lymphoid cells (ILC3), which in turn produce interleukin-22 (IL-22). IL-22 promotes the production of antimicrobial peptides by Paneth cells and IECs (15). Specifically, most of the blood flow from the small intestine and large intestine ultimately converges into the portal vein and enters the sinusoids of the liver. Here, sinusoidal endothelial cells activate Kupffer cells, which migrate to the periportal areas to protect the liver from pathogens and intestinal toxins such as trimethylamine (TMA), p-cresol (PC), and hydrogen sulphide (H_2_S) (16, 17).

The gut-liver axis functions as a bidirectional mechanism, indicating the presence of components that also transit from the liver to the gut (18). The components consist of bile, primarily made up of bile acids (BAs), IgA, antimicrobial peptides, and bicarbonate. This mixture demonstrates significant host defence properties. BAs are categorised as primary bile acids, which are conjugated with glycine or taurine by hepatocytes and subsequently secreted into bile through the bile salt export pump (BSEP). Secondary BAs undergo deconjugation by the gut microbiota (16). BAs offers antimicrobial benefits directly through its cleansing characteristics and simultaneously functions as an endogenous ligand for the farnesoid X receptor (FXR) by activating it. FXR is a nuclear receptor that plays a role in bile acid balance, glucose metabolism, insulin response, and the intestine innate immune response (19). In the ileum, BAs activate FXR, which initiates a negative feedback mechanism that inhibits the *de novo* synthesis of BAs in the liver, suppresses gluconeogenesis, activates glycogen synthesis in the liver, increases energy expenditure in muscle and brown adipose tissue, and induces insulin production in pancreatic β-cells (20). The activation of FXR by bile acids not only self-regulates their composition but also modulates various transcription factors associated with lipogenesis, inflammation, and fibrosis (21–23). IgA-producing plasma cells located in the lamina propria represent the main source of intestinal IgA. The polymeric immunoglobulin receptor (pIgR), expressed on the basolateral surface of epithelial cells, transports IgA across epithelial cells. At the apical surface, the IgA-pIgR complex is cleaved and IgA, bound to the extracellular portion of pIgR, is released as secretory IgA (sIgA) (24, 25). IgA deficiency or abnormal levels directly promote the progression of non-alcoholic fatty liver disease to non-alcoholic steatohepatitis by disrupting the epithelial barrier function of the gut-liver axis, exacerbating gut microbiota dysbiosis, and causing systemic immune disorders (26, 27).

Dysbiosis of the gut microbiota can result in hepatic metabolic abnormalities and liver injury; affected livers are incapable of efficiently eliminating detrimental metabolic byproducts, therefore hastening disease advancement. Research indicates that the gut-liver axis is implicated in the aetiology of various liver illnesses, including alcoholic liver disease, non-alcoholic steatohepatitis, acute and chronic liver failure, cirrhosis, and liver cancer (28, 29). The gut microbiota significantly affects the fundamental processes of host metabolism via the gut-liver axis, which is largely contingent upon the integrity of the intestinal barrier.

## 3 Intestinal barrier

The intestinal barrier is a multilayered defence system composed of mechanical, biological, chemical, and immunological components that cooperate to maintain intestinal homeostasis and protect the host from harmful luminal substances. The mechanical barrier, formed by IECs, tight junctions (TJs), and the mucus layer, provides the first physical line of defence (30, 31). Mucus secreted by goblet cells lubricates the intestinal surface, while Claudins, Occludin, and ZO-1 preserve epithelial integrity and limit the paracellular passage of pathogens, toxins, and macromolecules (32, 33). Disruption of these components is associated with increased intestinal permeability and systemic inflammation. The biological barrier is constituted by the resident gut microbiota, which suppresses pathogen colonisation through competitive exclusion, participates in immune regulation, and maintains ecological balance (34). Dysregulated microbiota may lead to a decline in intestinal barrier function, increase the risk of harmful microbial colonization, and even cause intestinal inflammation or other systemic diseases. FMT may help restore beneficial microbial communities (35, 36). The chemical barrier includes bile acids, digestive enzymes, antimicrobial peptides, and other bioactive secretions that not only support digestion and absorption but also shape microbial composition and mucosal immunity (37, 38). Disturbances in bile acid homeostasis may aggravate dysbiosis, while modulation of gut microbiota can reshape bile acid metabolism and increase beneficial secondary bile acids (39). The immune barrier, composed of sIgA, T cells, B cells, macrophages, and cytokines, provides the main immune defence of the gut. In particular, sIgA limits pathogen adhesion and promotes their clearance in mucus. It also supports immune tolerance to food antigens and commensal microbiota, which is essential for mucosal immune homeostasis (40, 41). Collectively, these four layers are functionally interconnected, and disruption of any component may impair barrier integrity, promote inflammation, and contribute to metabolic abnormalities.

## 4 Gut microbiota causes intestinal inflammation

The gut microbiota’s influence on the initiation and advancement of chronic inflammation has garnered significant interest. The gut microbiota is essential for gastrointestinal health and significantly impacts systemic chronic inflammation via its metabolic byproducts, immune regulatory roles, and interactions with the host immune system. Research has increasingly focused on the role of gut microbiota in chronic inflammation. Dysbiosis of the gut microbiota induces chronic low-grade inflammation via metabolic endotoxemia and alters the metabolism of short-chain fatty acids (SCFAs) and BAs, thereby affecting metabolic functions and promoting disease progression (42–44).

### 4.1 Gut microbiota dysbiosis and metabolic endotoxemia

Dysbiosis, or the disruption of the gut microbiota balance, is one of the core mechanisms of chronic inflammation (45, 46). Under normal conditions, the gut microbiota closely interacts with the intestinal barrier to maintain intestinal homeostasis (47). However, when the gut microbiota becomes imbalanced, especially with the proliferation of gram-negative bacteria like *Escherichia coli*, the integrity of the intestinal barrier may be compromised, leading to the leakage of endotoxins, such as lipopolysaccharides (LPS) (48, 49). LPS is a constituent of the cell wall in gram-negative bacteria and serves as a significant immune activator. LPS interacts with the CD14 receptor, activates Toll-like receptor 4 (TLR4), and subsequently triggers the downstream nuclear factor kappa-B (NF-κB) signalling pathway (50, 51). Following NF-κB activation, there is an increase in the production of pro-inflammatory cytokines, including TNF-α, IL-6, IL-1β. Cytokines serve as indicators of chronic low-grade inflammation (52).

In dysbiosis, patients with type 2 diabetes mellitus (T2DM) generally display an elevation of pathogenic bacteria (LPS-containing *Gram-negative bacteria*), while the population of beneficial bacteria that generate SCFAs, like butyrate, diminishes, resulting in impairment of the intestinal mucosal barrier and heightened intestinal permeability (53). Elevated levels of LPS penetrate the bloodstream through the intestinal barrier and disseminate to multiple organs via the circulatory system, thereby intensifying chronic inflammatory responses. The deficiency of SCFAs results in elevated intestinal pH, which facilitates the growth of opportunistic pathogens, including *Desulfovibrio*, and diminishes the expression of tight junction proteins, such as ZO-1 and occludin, in the intestinal barrier, thereby worsening endotoxemia (54, 55).

### 4.2 Gut microbiota and insulin resistance

Chronic low-grade inflammation is a significant contributor to the onset of type 2 diabetes. The chronic inflammatory condition impairs insulin action by the secretion of pro-inflammatory cytokines, resulting in insulin resistance. Insulin resistance is intimately associated with elevated inflammatory markers, particularly LPS, which can directly obstruct insulin signalling by activating immune receptors (TLR4) and the NF-κB pathway, so exacerbating inflammation in adipose tissue and the liver (56–58). Furthermore, the LPS-activated TLR4/NF-κB pathway can stimulate the generation of reactive oxygen species (ROS), resulting in pancreatic β cell malfunction and hepatic cellular damage, hence aggravating the dysregulation of glucose and lipid metabolism (59). Moreover, LPS intensifies the development of insulin resistance by compromising the intestinal barrier and enhancing intestinal permeability, so facilitating the entry of additional metabolic endotoxins into the bloodstream (60–62). Studies have shown that obese and T2DM patients have higher levels of LPS and other endotoxins in their intestines, which spread throughout the body, activating immune cells such as macrophages and adipocytes, and prompting them to secrete more pro-inflammatory cytokines (63, 64). These cytokines not only exacerbate local inflammatory responses but also affect target tissues such as the liver and muscles through the bloodstream, leading to reduced insulin receptor sensitivity to insulin, ultimately resulting in insulin resistance.

### 4.3 The protective effect of SCFAs and the intestinal barrier

SCFAs, especially butyrate, propionate, and acetate, are metabolites generated by gut probiotics via the fermentation of dietary fibres, significantly influencing gut health and the systemic immune system (65). In a typical gut microecological environment, SCFAs contribute to the maintenance of IECs function and promote gut barrier integrity, while also modulating the systemic inflammatory response via their effects on the immune system (66, 67). SCFAs interact with G protein-coupled receptors (GPCRs), which activates anti-inflammatory signalling pathways and suppresses the NF-κB pathway, leading to a decrease in pro-inflammatory cytokine release (68–70). Research indicates that SCFAs can enhance the secretion of IL-10, a significant anti-inflammatory agent that effectively mitigates inflammation triggered by LPS (71).

Gut microbiota imbalance results in a decrease in probiotic populations, leading to diminished SCFA production, gut barrier disruption, and sustained chronic low-grade inflammation. Insufficient levels of SCFAs facilitate the proliferation of detrimental gut microbiota, resulting in increased production of LPS, which exacerbates metabolic endotoxemia and ultimately contributes to insulin resistance and the onset of T2DM (72).

### 4.4 Bile acid metabolism and chronic inflammation

In addition to SCFAs, BAs are significant metabolites produced by the liver and modified by the gut microbiota. BAs are essential for the digestion and absorption of fats, and they also modulate the gut barrier and immune response through interactions with the gut microbiota (73, 74). Studies have shown that gut microbiota imbalance may lead to abnormal bile acid metabolism, thereby affecting the synthesis and metabolism of BAs, which in turn impacts gut health and overall metabolism (21).

**Figure 1:**
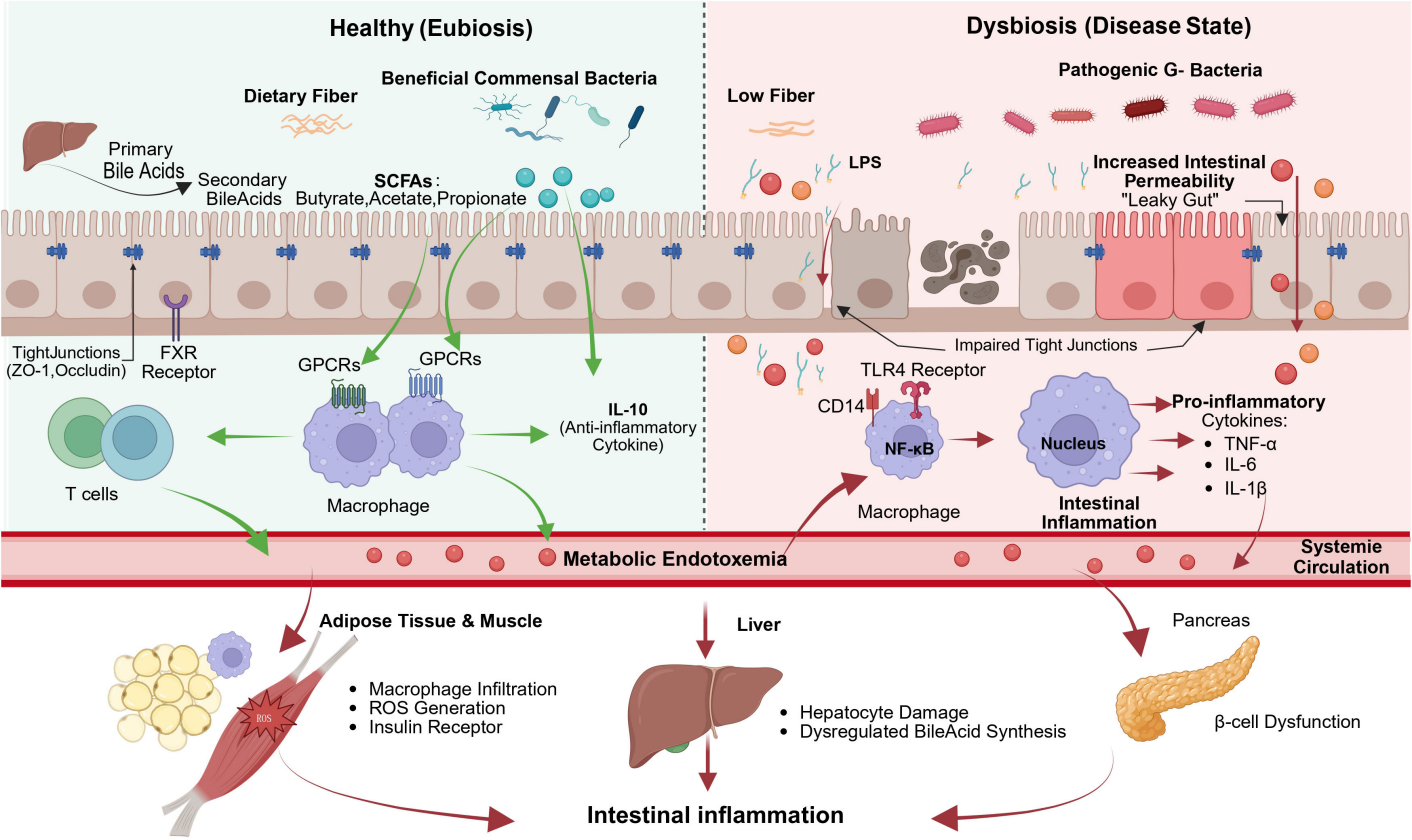
Mechanisms of gut microbiota-induced intestinal inflammation and systemic insulin resistance. In the healthy state, dietary fibre fermentation by beneficial bacteria produces SCFAs, which strengthen intestinal tight junctions and exert anti-inflammatory effects via GPCRs and IL-10. FXR activation by bile acids further supports barrier integrity. In dysbiosis, reduced fibre intake promotes pathogenic Gram-negative bacteria, which release LPS. LPS disrupts tight junctions, increases gut permeability, and activates the CD14/TLR4/NF-κB pathway in macrophages, leading to pro-inflammatory cytokine release (TNF-α, IL-6, IL-1β). These factors enter circulation, causing metabolic endotoxemia, which impairs insulin signalling in adipose tissue and muscle, dysregulates bile acid synthesis in the liver, and damages pancreatic β-cells—collectively driving systemic insulin resistance and T2DM progression.

BAs are crucial in regulating fat metabolism, bile acid synthesis, and immune response through the activation of nuclear receptors, including the FXR (75). The activation of the FXR receptor can modulate the metabolic condition of the liver and intestine, enhance gut barrier integrity, and mitigate intestinal inflammation (76). An imbalance in gut microbiota can disrupt bile acid metabolism, influencing metabolic pathways, which in turn exacerbates gut microbiota imbalance and chronic inflammation, ultimately facilitating the formation of T2DM (77, 78). The mechanisms by which gut microbiota dysbiosis drives intestinal inflammation and systemic insulin resistance are summarized in **Figure 1**.

## 5 Changes in the microbiota of patients with GLMD

Dysbiosis of the gut microbiota is strongly linked to the development of numerous metabolic disorders. The gut microbiota of patients with various disorders displays distinct alterations, frequently encompassing particular microbial communities, metabolic byproducts, and interactions with host immunity and metabolic processes.

### 5.1 The relationship between obesity and gut microbiota

Numerous research have substantiated the correlation between obesity and the gut flora. The gut microbiota diversity in obese people is markedly reduced compared to that in healthy persons, and its composition has experienced considerable alterations (79, 80). In obese individuals, the *Firmicutes* to *Bacteroidetes* ratio is elevated, and the prevalence of specific opportunistic infections, such as *Escherichia coli*, is enhanced (81). Specifically, the abundance of probiotics such as *Lactobacillus* and *Bifidobacterium* is negatively correlated with the degree of obesity, while the abundance of certain harmful bacterial groups (*Enterococcus*, *Bacteroides*, and *Clostridium*) increases (82, 83). The production of SCFAs, especially butyrate, decreases, which may be closely related to the decline in gut barrier function, enhanced inflammatory response, and increased energy intake (84–86). Additionally, the dysbiosis of the gut microbiota in obese patients promotes the onset and progression of obesity by affecting appetite regulation, energy absorption, inflammatory response, and circadian rhythm control (87, 88).

### 5.2 The relationship between abnormal blood sugar levels and gut microbiota

Patients with prediabetes and diabetes demonstrate reduced diversity in gut bacteria. The microbial diversity in prediabetic patients is markedly diminished compared to that in healthy persons, characterised by a decrease in 9 genera and an increase in 14 genera (89), among which T2DM is closely associated with gut microbiota dysbiosis. In T2DM patients, the gut microbiota typically exhibits an increased abundance of certain harmful bacterial groups (*Escherichia coli*, *Clostridium*, and other opportunistic pathogens), while the abundance of beneficial bacteria is reduced. This includes some *Bifidobacterium* species (*B. adolescentis, B. bifidum, B. pseudocatenulatum, B. longum, B. dentium*) and some *Bacteroides* species such as *Bacteroides intestinalis*, *Bacteroides* 20-3, and *Bacteroides vulgatus* (90–92). The deficiency of SCFAs is considered one of the important pathogenic mechanisms of T2DM. SCFAs not only help control blood glucose by regulating appetite and enhancing insulin sensitivity but also maintain gut barrier function to prevent endotoxin entry (93, 94). The increase of metabolic endotoxins such as LPS can cause chronic low-grade inflammation, leading to the onset of insulin resistance (95). Furthermore, long-term use of statins (atorvastatin) disrupts the expression of gut tight junction proteins, increasing intestinal permeability, facilitating the entry of LPS into the bloodstream, and activating the NF-κB inflammatory pathway, ultimately triggering chronic metabolic inflammation and disruption of blood glucose homeostasis, associated with an increase in *Oscillibacter* and a decrease in *Akkermansia* (96). Alterations in the gut microbiota may exacerbate the advancement of diabetes by modifying bile acid metabolism, levels of branched-chain amino acids, and immunological responses (97).

### 5.3 The relationship between dyslipidaemia and gut microbiota

Dyslipidaemia refers to abnormal serum lipid levels and has become a major cause of increased morbidity and mortality worldwide (98). The aetiology is multifaceted, involving genetic, behavioural, and environmental variables, with gut microbial dysbiosis also significantly contributing (99). Research indicates that the composition of gut microbiota is intricately linked to cholesterol metabolism and dyslipidaemia. Transplantation of gut microbiota from dyslipidemic patients into animals can enhance intestinal cholesterol absorption and result in hypercholesterolaemia (100). Additionally, studies using germ-free mouse models have found that the gut microbiota plays a crucial role in fat intake and lipid metabolism, with germ-free mice having lower triglyceride and low-density lipoprotein (LDL) levels and being resistant to diet-induced obesity (101). Human studies indicate that SCFA-producing bacteria in the gut microbiota (*Akkermansia*, *Bacteroides*, and *Roseburia*) are less abundant in patients with dyslipidaemia, while harmful bacteria (*Escherichia coli* and *Enterobacter*) show increased concentrations (102, 103). These alterations signify the regulatory function of the gut microbiota in lipid metabolism and may affect blood lipid concentrations by modifying metabolic byproducts (SCFAs). For example, SCFAs promote the conversion of cholesterol to BAs and inhibit the activity of key liver lipid synthesis enzymes (HMGCR and FAS), thereby reducing serum total cholesterol and low-density lipoprotein levels (104–106).

### 5.4 The relationship between MAFLD and gut microbiota

MAFLD is a disease associated with insulin resistance, hepatic fat accumulation, and chronic inflammation (13, 107). The onset of MAFLD is closely related to gut microbiota dysbiosis, and the gut microbiota characteristics of patients are significantly different from those of healthy individuals. The gut microbiota dysbiosis in MAFLD patients is typically manifested by changes in the abundance of *Bacteroidetes* and *Firmicutes* phyla. An increase in *Bacteroidetes* and a decrease in *Firmicutes* are closely associated with the development and progression of MAFLD (108, 109). In adolescent MAFLD patients, the abundance of *Clostridium difficile* and *Salmonella* spp. in the gut increases, while *Bifidobacterium* and *Prevotella* are more abundant, and *Lactobacillus* is significantly reduced (110); in non-obese MAFLD patients, the abundance of *Proteobacteria* increases, while *Lactobacillus* and *Dubosiella* are reduced (111), and changes in the abundance of *Faecalibacterium*, *Roseburia*, and other genera can distinguish MAFLD subtypes (112). This microbiota imbalance affects liver metabolism through multiple pathways of the gut-liver axis: intestinal barrier damage leads to leakage of endotoxins (LPS), activating liver inflammation and insulin resistance (113, 114); reduction in SCFAs production and abnormalities in metabolites such as trimethylamine N-oxide (TMAO) exacerbate lipid metabolic disorders (115–117); disruption of bile acid metabolism interferes with lipid homeostasis through the FXR/TGR5 signalling pathway (118, 119); and a reduction in specific microbiota, such as Akkermansia, promotes liver fibrosis through immune dysregulation (120, 121). These mechanisms together form the core network through which the gut microbiota regulates the pathological progression of MAFLD.

Dysbiosis of gut microbiota compromises the intestinal barrier’s integrity, resulting in the significant influx of endotoxins (LPS) into the bloodstream, which then reach the liver via the portal vein, inciting liver inflammation. Metabolites from the gut microbiota, especially short-chain fatty acids and bile acids, are critical in the pathogenesis of MAFLD (122). A decrease in SCFAs and disturbance of bile acid metabolism accelerates the course of MAFLD, whereas normal levels of these metabolites promote gut health and limit hepatic fat storage.

### 5.5 The relationship between atherosclerosis and gut microbiota

The pathogenesis of atherosclerosis (AS) is complex, involving factors such as endothelial damage, platelet aggregation, inflammatory responses, and lipid metabolism disorders (123). Recent research indicate that gut microbiome significantly influences the onset and progression of AS (124, 125). Studies demonstrate that gut microbiota and its metabolites significantly influence the initiation and regulation of systemic inflammatory reactions and can alter risk factors for diverse cardiovascular illnesses (126, 127). The gut microbiota regulates systemic inflammatory responses by maintaining the integrity of the intestinal barrier and preventing pathogens and their metabolites from entering the bloodstream (128). An imbalance in gut microbiota compromises intestinal barrier function, permitting pathogen-associated molecular patterns (PAMPs) to enter the bloodstream, activate the immune system, and contribute to chronic low-grade inflammation, a significant factor in the development of AS. Studies have found that although the gut microbiota diversity in AS patients is similar to that of healthy individuals, certain bacterial abundances significantly change, such as a reduction in *Prevotella* and *Bacteroides*, which may impair immune function and promote inflammatory responses (129). At the same time, the reduction of *Lachnospiraceae* and the increase in *Rothia* are associated with enhanced inflammation and immune responses (130). Lower abundance of Lachnospiraceae NK4B4 group, Lachnospiraceae UCG-004, and *Ruminococcus gauvreauii*, and higher abundance of *Ruminococcus gnavus* were significantly associated with coronary artery disease (131). In addition, metabolic products of the gut microbiota, such as BAs, may play an important role in the development of AS. The microbiome affects reverse cholesterol transport by modulating bile acid metabolism, including the inhibition of the FXR signalling pathway (132, 133). Meanwhile, studies have found that the gut microbiota regulates host inflammatory responses, lipid metabolism, and endothelial function through metabolites such as TMAO and SCFAs, affecting plaque formation and stability (134, 135). TMAO is produced by hepatic oxidation of trimethylamine (TMA), which is derived from the gut bacterial metabolism of dietary choline and L-carnitine. TMAO can promote macrophage foam cell formation, enhance platelet activity, and increase vascular inflammation (136, 137), while SCFAs (e.g., butyrate) reduce inflammation and enhance intestinal barrier function and vascular function by inhibiting histone deacetylases (HDAC) (135, 138). The findings indicate that alterations in the makeup of gut microbiota and its metabolites may significantly influence the onset and progression of AS.

## 6 FMT treatment for GLMD

**Figure 2:**
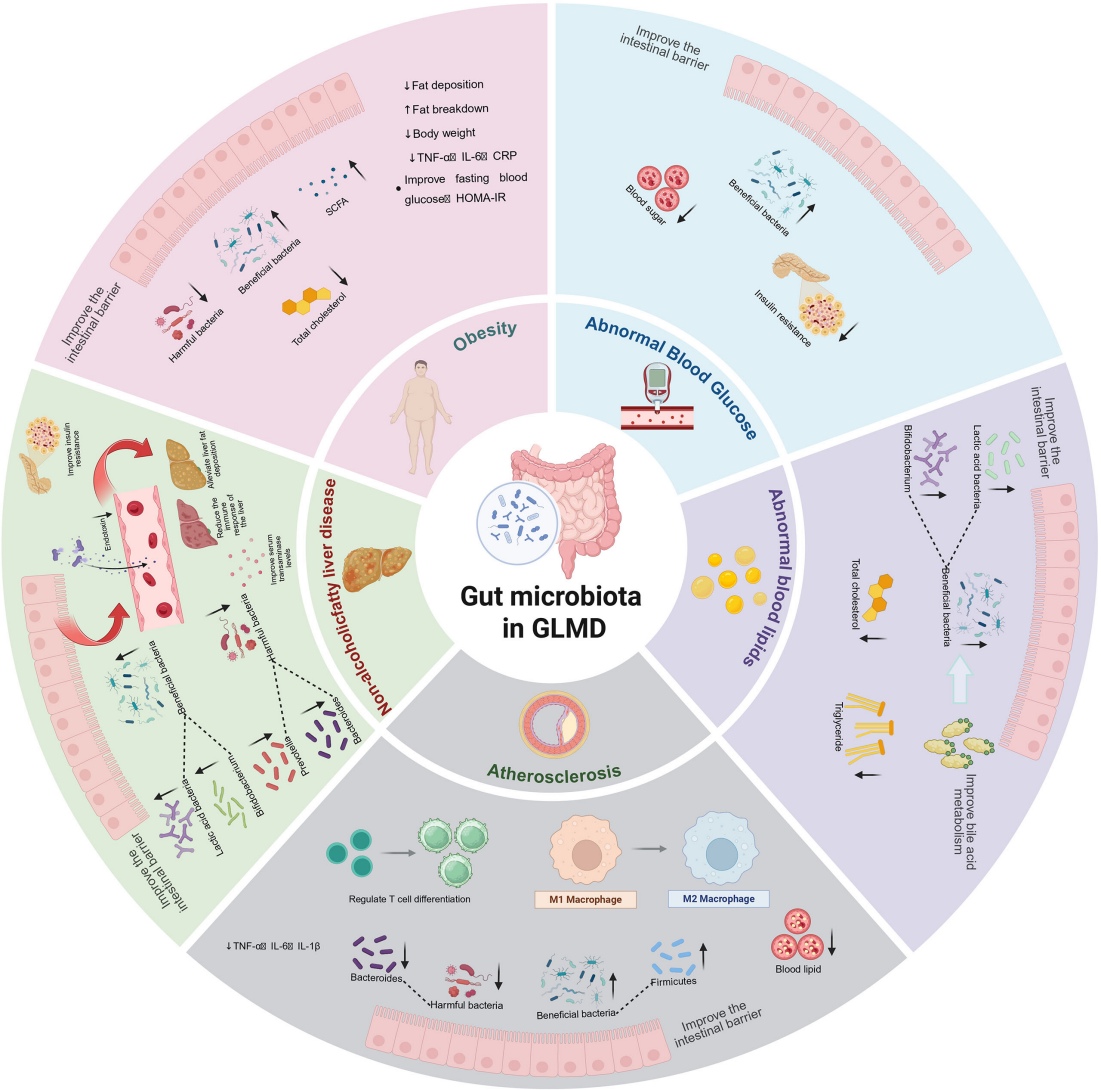
The interaction between GLMD and gut microbiota. Gut microbiota influences GLMD by enhancing lipid metabolism and insulin sensitivity, reducing fat accumulation, hepatic steatosis, and inflammation. It improves glucose and lipid homeostasis, regulates bile acid metabolism, and modulates immune responses, thereby alleviating obesity, dyslipidaemia, MAFLD, and atherosclerosis.

FMT, an emerging therapeutic method, has steadily shown its potential in the treatment of metabolic illnesses related to glucose and lipid metabolism, especially in obesity, metabolic syndrome (MetS), T2DM, and dyslipidaemia, displaying positive effects in recent years (139–142). FMT restores the balance of the gut microbiota, improves microbial diversity, and thus affects the metabolic functions of the host (143, 144). Although FMT has demonstrated good efficacy in short- and mid-term studies, its safety concerns warrant more investigation, particularly in risk evaluations for specific populations and long-term follow-up. **Figure 2** shows how gut microbiota can improve GLMD.

### 6.1 Obesity

Patients with obesity and metabolic syndrome often exhibit dysbiosis of the gut microbiota; however, the overall efficacy of FMT in these conditions remains inconsistent across population-based studies. Several randomized controlled trials (RCTs) and systematic reviews suggest that FMT may improve certain metabolic parameters, such as fasting blood glucose, the homeostasis model assessment of insulin resistance (HOMA-IR), total cholesterol, and inflammatory markers to some extent; however, its effect on weight loss is limited or inconsistent (145–147). A 2024 meta-analysis incorporating 14 RCTs and a total of 578 participants further demonstrated that FMT did not significantly reduce body weight within 6 months, nor did it consistently improve metabolic parameters such as lipid profiles and insulin sensitivity (148) Although the overall evidence remains insufficient, potential benefits have been observed in certain specific populations. For example, a 4-year randomized double-blind controlled trial indicated that FMT combined with dietary fibre supplementation improved insulin sensitivity in adolescents with severe obesity and metabolic syndrome, significantly reducing HOMA-IR, fasting blood glucose, and lipid levels, though weight changes were not significant; These metabolic improvements were associated with increased abundances of beneficial bacteria such as *Bifidobacterium* and *Akkermansia* (149). Therefore, as of the end of 2025, there is insufficient evidence to support FMT as a long-term effective treatment for obesity. Future studies requiring larger sample sizes and longer follow-up periods are needed to clarify its role in obesity management and to explore optimized strategies for its combined use with dietary interventions or probiotic supplementation.

In terms of safety, FMT is generally well-tolerated in patients with obesity and metabolic syndrome, with few short-term adverse reactions, primarily manifested as mild gastrointestinal discomfort (150). However, some patients may experience significant gastrointestinal dysfunction following FMT, which may be related to the interaction between the donor microbiota and the recipient’s native microbiota (150). Furthermore, for patients with compromised immune function—such as those with immunodeficiency or other chronic diseases—caution is still warranted regarding risks such as pathogen transmission, abnormal immune responses, and metabolic disturbances (151–153). Therefore, in these special populations, donor screening should be strengthened, and post-treatment monitoring should be carefully conducted.

Animal and mechanistic studies support the potential role of FMT in obesity. Research indicates that obesity and metabolic syndrome are often accompanied by gut dysbiosis, and that FMT can improve the composition of the gut microbiota by reducing the proportion of harmful bacteria and increasing beneficial bacteria such as Bifidobacterium and Lactobacillus, thereby improving fat metabolism and insulin sensitivity (154). A study by Zhu X et al. showed that FMT can reverse microbiota dysbiosis in obese individuals by restoring a healthy gut microbiome, improve intestinal barrier function, reduce metabolic inflammation, and ultimately reduce abdominal fat deposition (146). In mice with high-fat diet-induced obesity, FMT led to increased abundance of short-chain fatty acid-producing bacteria such as *Akkermansia muciniphila*, accompanied by upregulation of genes associated with lipolysis in adipose tissue (146, 154). Multiple animal studies have also demonstrated that faecal microbiota transplantation from lean donors to obese animals significantly reduces body weight and improves glucose tolerance and fatty liver (155–157). These findings suggest that FMT may primarily exert its effects by reshaping the gut microbiota, improving intestinal barrier function, and regulating host metabolic pathways. To provide a comprehensive overview of the current evidence, **Table 1** summarizes key findings from recent clinical trials and animal models. This table highlights the specific microbiota changes, metabolic outcomes, and safety profiles associated with FMT interventions in obesity.

**Table 1:** Summary of clinical trials and animal studies evaluating faecal microbiota transplantation in obesity management.

Study setting and model	FMT protocol (donor source + administration route)	Key microbiota changes after FMT	Main metabolic outcomes	BA/SCFA changes	Safety	Ref.
Clinical studies						
12-week double-blind RCT; adults with obesity and mild-to-moderate insulin resistance (n=24)	Donor: lean healthy donors; route: oral capsules; regimen: randomized 1:1 *vs* placebo for 6 weeks.	Higher Prevotellaceae/*Bacteroidaceae* ratio	No significant change in HOMA-IR, body weight, or body composition	—	Mild diarrhoea was more frequent in the FMT group	(158)
Randomized controlled clinical trial; obesity cohort (n=75 total)	Donor: healthy university-student donors; route: not reported; comparator: non-FMT control received oral probiotics.	Increased Selenomonadales, Veillonellaceae, Prevotella 2 and uncultured Roseburia; decreased Ruminococcus gnavus group and *Bacteroides*	Reduced liver fat deposition; clearer benefit in the lean subgroup	—	No significant change in serum lipids or liver function tests	(159)
Randomized double-blind placebo-controlled trial; adults with obesity and mild-to-moderate insulin resistance (n=22)	Donor: lean healthy donors; route: oral capsules.	Engraftment of *Faecalibacterium*-related taxa	No significant reduction in BMI; no improvement in GLP-1 AUC	Lower faecal taurocholic acid; BA profile shifted toward donor profile	Well tolerated	(160)
Double-blind randomized placebo-controlled trial; adolescents with obesity (n=87)	Donor: four sex-matched lean donors; route: acid-resistant oral capsules.	Decreased *Escherichia coli*; increased Faecalibacterium prausnitzii, Bacteroides ovatus and Alistipes spp.	No significant change in BMI SDS; more remission in the metabolic syndrome subgroup by week 26	—	Only mild gastrointestinal symptoms were reported	(161)
Double-blind randomized trial; obesity intervention study (n=70)	Donor: lean/healthy donors; route: oral capsules; co-intervention: high- or low-fermentable-fibre diet.	Increased Phascolarctobacterium, Christensenellaceae, Bacteroides and *Akkermansia muciniphila*; decreased Dialister and Ruminococcus torques	Improved HOMA2-IR in the FMT + low-fermentable-fibre group	No significant change in faecal SCFAs	No treatment-related serious adverse events	(162)
Animal studies						
HFD-induced obesity model; male C57BL/6N mice (n=48)	Donor: wild-boar faecal suspension; route: not reported; regimen: low- and high-dose FMT.	Increased Lactobacillus and Romboutsia, especially in the high-dose FMT group	Improved GTT/ITT; lower glucose, TG and total cholesterol, although body weight increased	—	—	(154)
Diet-induced obesity model; male Wistar rats (n=40)	Donor: low-fat-diet rat cecal contents; route: not reported; recipients: HFD + antibiotic-treated rats.	Bacteroidetes and Firmicutes shifted toward the low-fat donor profile	Body weight and fat mass rebounded after FMT following antibiotic-induced decline	—	—	(163)
Human-to-mouse transfer study; germ-free C57BL/6J mice (n=103)	Donor: lean *vs* obese human twins; route: not reported; recipients: germ-free mice on low-fat diet.	Lean-donor taxa such as Bacteroides uniformis, B. vulgatus and B. cellulosilyticus invaded and corrected the obese configuration	Improved hepatic/skeletal muscle acylcarnitine abnormalities and mild glucose intolerance under permissive diet conditions	Higher butyrate and propionate with lean-donor microbiota	—	(164)
Exercise/diet donor microbiota to HFD-fed obese rodents (n=8/group)	Donor: exercised or diet-matched donors; route: oral gavage.	Restored Bacteroidetes and Firmicutes and improved Helicobacter, Odoribacter and AF12	Reduced body weight gain, fat mass and food efficiency; improved fasting glucose, IPGTT, ALT and LDL	—	—	(165)
Healthy-donor transfer to abdominal-obesity model; C57BL/6J mice (n=5/group)	Donor: fresh feces from healthy age-matched mice; route: oral gavage.	Increased Akkermansia	Reduced body-weight gain, waist circumference and abdominal/liver fat; improved glucose tolerance	—	—	(146)

### 6.2 Abnormal blood glucose

The occurrence of T2DM is closely related to gut microbiota dysbiosis. FMT regulates the composition of gut microbiota, improves gut barrier function, and reduces metabolic inflammation, showing potential therapeutic effects for T2DM (166). Research demonstrates that faecal microbiota transplantation can markedly enhance insulin resistance and glycaemic regulation in newly diagnosed T2DM patients (167). The research indicated that FMT can modulate gut microbiota composition, enhance the prevalence of beneficial bacteria, improve insulin sensitivity, and facilitate the normalisation of blood glucose levels. When administered alongside metformin, FMT demonstrates enhanced efficacy, indicating that FMT functions not only independently in mitigating insulin resistance but also exhibits a synergistic impact with traditional pharmacotherapies. Furthermore, FMT exhibits control of the interplay between gut microbiota and glucose metabolism. Research indicates that in certain T2DM patients, FMT results in changes in specific bacterial strains in the gut microbiota, including the families *Ruminococcaceae* and *Rikenellaceae*. In non-responders, the abundances of *Rikenellaceae* and *Anaerotruncus* before and after treatment remain similar to those observed in healthy controls, whereas in responders FMT adjusts their abundance toward levels observed in healthy individuals (168). FMT improves the symptoms of diabetes by restoring the balance of these bacteria. A 24-week randomized controlled trial demonstrated that FMT effectively restored the gut microbiota in obese patients with type 2 diabetes by increasing beneficial bacteria (particularly butyrate-producing bacteria), improving microbial diversity, and enhancing metabolic products. Combining FMT with lifestyle interventions further enhanced microbial colonization and metabolic improvements (169).

FMT is generally safe in patients with T2DM (170). Most studies have not shown significant differences in insulin, blood glucose, weight, and other parameters between the FMT group and the placebo group, but some studies suggest that FMT has a noticeable improvement effect in certain patients (158, 171–173). These differences may be related to individual differences, variations in gut microbiota composition, and differences in experimental design. While FMT is generally safe for the majority of T2DM patients, its safety in older individuals, those with severe complications, or patients with gastrointestinal disorders requires additional scrutiny. These patient populations exhibit diminished immune functioning and may be more vulnerable to harmful effects. Consequently, while administering FMT in these specific patient populations, it is imperative to ascertain the health state of the donor microbiota and implement rigorous monitoring. A detailed compilation of recent clinical trials and animal experiments evaluating the impact of FMT on glycaemic control, insulin resistance, and microbiota reconstruction is presented in **Table 2**.

**Table 2:** Overview of clinical and preclinical studies on faecal microbiota transplantation for glycaemic control and type 2 diabetes mellitus.

Study setting and model	FMT protocol (donor source + administration route)	Key microbiota changes after FMT	Main metabolic outcomes	BA/SCFA changes	Safety	Ref.
Clinical studies						
Prospective open-label single-arm study; T2DM patients (n=17) with healthy controls (n=20)	Donor: healthy human donors; route: not reported; regimen: two FMT procedures within 2 days.	Rikenellaceae and Anaerotruncus shifted toward healthy levels	Lower fasting/postprandial glucose, HbA1c and uric acid; higher postprandial C-peptide; no clear change in body weight/BMI	—	No significant FMT-related adverse events or gastrointestinal symptoms	(168)
Prospective exercise intervention with mouse FMT validation; untreated prediabetic men and obese mice	Donor: responder *vs* non-responder post-exercise human microbiota; route: mouse gavage.	Decreased Alistipes shahii, Alistipes putredinis and Ruminococcus gnavus	Improved fasting/fed glucose, insulin, GTT and ITT in obese recipient mice	Circulating SCFAs increased in mice receiving responder-after-exercise microbiota	—	(95)
90-day open-label controlled trial; T2DM patients (n=16)	Donor: healthy human donors; route: oral capsules; co-intervention: diet.	Shift from Bacteroides-dominant to Prevotella-dominant profile; increased Bifidobacterium and Lactobacillus; decreased Bilophila and Desulfovibrio	BMI and HbA1c decreased	—	No abdominal pain or diarrhoea reported	(174)
Randomized placebo-controlled trial; obesity with T2DM (n=61)	Donor: healthy human donors; route: not reported; co-intervention: lifestyle intervention in one arm.	Higher Bifidobacterium and Lactobacillus in the FMT + lifestyle group	Improved lipid profile and liver stiffness; lower total lipoprotein and LDL-C	More butyrate-producing bacteria after FMT	—	(169)
Randomized controlled prospective study; newly diagnosed T2DM (n=29 completed)	Donor: healthy human donors; route: nasojejunal infusion to the proximal jejunum; with or without metformin.	Increased Prevotella jejuni, Prevotella fusca, Bifidobacterium animalis and B. adolescentis	FMT alone and FMT + metformin reduced HOMA-IR and BMI; FMT alone also improved FBG, PBG, HbA1c and HOMA-HBCI; combination additionally reduced TG and uric acid	Total bile acids decreased after FMT	No adverse reactions, hypoglycaemia or dyslipidaemia	(167)
Phase II randomized single-blind parallel trial; T2DM patients (n=21)	Donor: two lean healthy donors; route: freeze-dried oral capsules; regimen: single dose.	Greater donor-species engraftment with reduced recipient species	No significant change in weight, BMI, HbA1c, OGTT glucose/insulin or HOMA-IR	—	No mild or severe treatment-related adverse events	(175)
Animal studies						
Therapeutic FMT in db/db mice (15 db/m donors; 27 db/db recipients)	Donor: KNGT microbiota donor; route: oral gavage for 10 weeks.	Decreased Desulfovibrio and Clostridium coccoides; increased *Akkermansia muciniphila*	Lower FBG, PPG, TC, TG and LDL-C; higher HDL-C	—	—	(176)
Mechanistic FMT study in db/db mice (n=9/group)	Donor: KNGT microbiota donor; route: oral administration.	Increased Bacteroides uniformis and Clostridium; decreased Mucispirillum schaedleri	Improved glucolipid metabolism; higher GPR43 mRNA and GLP-1 protein	Acetate and butyrate increased	—	(177)
Therapeutic FMT in HFD + STZ-induced T2DM mice (n=4-8/group)	Donor: healthy mouse donors; route: intragastric gavage for 8 weeks.	—	Lower FBG, HbA1c and HOMA-IR; improved OGTT, HOMA-IS and HOMA-beta; reduced inflammation and beta-cell apoptosis	—	—	(178)
Therapeutic FMT in db/db mice (16 recipients)	Donor: fresh faecal suspension from db/m healthy donors; route: oral gavage for 4 weeks.	Increased Ruminococcaceae and Porphyromonadaceae; decreased Rikenellaceae and Lactobacillaceae; higher Bacteroidetes/Firmicutes ratio	Improved OGTT and HOMA-IR; reduced fasting glucose and insulin; restored small-intestinal barrier	—	—	(179)
Therapeutic FMT in BTBR ob/ob mice with DKD (n=6/group)	Donor: healthy BTBR wild-type donors; route: rectal administration.	Increased Odoribacteraceae	Suppressed weight gain, reduced albuminuria/UACR and improved HOMA-IR-related phenotypes	—	No structural damage to intestinal epithelium or tight junctions	(180)

### 6.3 Abnormal blood lipids

FMT demonstrates efficacy in the management of dyslipidaemia, particularly in enhancing total cholesterol and triglyceride levels (181–184). Research indicates that FMT can improve the lipid profile by adjusting the composition of gut microbiota and increasing the abundance of beneficial bacteria, such as lactobacilli and bifidobacteria (174). FMT can significantly reduce total cholesterol and triglycerides, with more pronounced effects when combined with dietary interventions. Some studies have found that FMT improves bile acid metabolism, promotes the growth of beneficial bacteria, enhances gut barrier function, and ultimately improves lipid metabolism (160, 169). Randomized controlled trial demonstrated that FMT, through increasing beneficial microbiota (particularly butyrate-producing bacteria), improving microbial diversity and metabolites, combined with lifestyle interventions, can reduce total LDL cholesterol and liver stiffness (169). After FMT, the bile acid content in patients significantly decreases, which is associated with changes in the donor microbiota, further demonstrating the close relationship between gut microbiota and lipid metabolism.

Studies on the safety of FMT in dyslipidaemia suggest that it is generally safe; however, some research indicates that short-term adverse reactions may occur in specific patients (185). These adverse reactions are usually temporary and gradually disappear as the gut microbiota stabilizes. During FMT treatment, patients with intestinal inflammation or immune dysfunction may exhibit an elevated risk of infection (186). Consequently, for high-risk patients, meticulous donor screening and rigorous compliance with safety protocols are essential to prevent adverse outcomes (187). For a systematic overview of how FMT modulates lipid profiles, bile acid metabolism, and gut microbiota composition in both clinical and preclinical settings, refer to **Table 3**.

**Table 3:** Summary of faecal microbiota transplantation interventions and lipid profile outcomes in dyslipidaemia.

Study setting and model	FMT protocol (donor source + administration route)	Key microbiota changes after FMT	Main metabolic outcomes	BA/SCFA changes	Safety	Ref.
Clinical studies						
db/db mouse study (15 db/m donors; 27 db/db recipients)	Donor: healthy human microbiota; route: oral gavage to db/db mice.	Decreased Desulfovibrio and Clostridium coccoides; increased *Akkermansia muciniphila*	Lower FBG, PPG, TC, TG and LDL-C; higher HDL-C	—	—	(176)
Human-to-mouse transfer study; Apoe-/- and LDLr-/- recipients	Donor: normocholesterolaemic *vs* high-cholesterol/CVD-risk human donors; route: oral gavage of bacterial suspension.	High-cholesterol donor microbiota enriched Betaproteobacteria, Alistipes, Bacteroides and Barnesiella	Recipients of high-cholesterol donor microbiota developed higher plasma cholesterol and lower hepatic LDLr	Bile acid synthesis increased after microbiota depletion	—	(100)
Human dyslipidaemia microbiota transfer to mice	Donor: dyslipidaemic *vs* healthy human donors; route: oral gavage.	Higher Firmicutes/Bacteroidetes ratio; increased Faecalibaculum, Ruminococcaceae UCG-010, Negativibacillus and Fusobacterium; decreased Muribaculum and Parasutterella	Combined with HFD, dyslipidaemic-donor microbiota increased TC, TG, LDL-C, HDL-C, body weight and liver steatosis	CA, CDCA and DCA increased, whereas intestinal HDCA decreased	—	(188)
Therapeutic FMT validation in HFD mice (n=5/group)	Donor: plant-sterol-treated donor mice; route: gavage for 1 week.	Lower Lactobacillus, Lactococcus, Streptococcus, Bacillus and Allobaculum in donor-associated profile	Reduced TC, TG and LDL-C; increased HDL-C	—	—	(189)
Therapeutic FMT validation in mice (4 groups, n=10/group)	Donor: Monascus-fermented-ginseng-treated donor mice; route: oral gavage.	Restored Firmicutes/Bacteroidetes ratio; increased Oscillospiraceae, Bacillaceae and Lactobacillaceae	Improved lipid profile, reduced body weight and liver fat, enhanced cholesterol excretion and fatty-acid beta-oxidation	Promoted conversion of primary to secondary bile acids	—	(190)
Mechanistic mouse study (n=18)	Donor: healthy *vs* hyperlipidaemic mouse microbiota; route: oral gavage for 4 weeks.	—	Lower serum TC, TG and LDL; increased hepatic integrin alpha4+ ILC3s and total ILC3s	—	—	(191)

### 6.4 Metabolic associated fatty liver disease

Animal experiments have confirmed the therapeutic effect of FMT on MAFLD, mainly reflected in regulating gut microbiota, reducing liver fat degeneration-related indicators, and improving insulin sensitivity (192–197). Research indicates that patients with MAFLD exhibit reduced gut microbiota diversity, characterised by a decrease in beneficial bacteria, including *bifidobacteria* and *lactobacilli*, alongside an increase in detrimental bacteria such as *Prevotella* and *Bacteroides* (156). Meanwhile, the gut barrier decreases the entry of endotoxins into the bloodstream, which can diminish liver immune responses, alleviate MAFLD-related inflammation, and so ameliorate hepatic fat accumulation (159, 198). The metabolic products of the gut microbiota can influence fat metabolism and improve insulin sensitivity, thereby helping to alleviate MAFLD symptoms. Animal studies have found that FMT can restore the balance of gut microbiota, promote the growth of beneficial bacteria, thereby improving intestinal function and reducing liver inflammation and steatosis (199). FMT can significantly improve liver steatosis and serum transaminase levels in a MAFLD mouse model, and enhance insulin sensitivity (200). Comparison between single-donor (SD-FMT) and multi-donor (MD-FMT) shows that MD-FMT, with higher microbiota diversity, has a greater advantage in improving insulin resistance and liver fat degeneration (201).

Although existing clinical studies provide positive evidence of efficacy, it is necessary to identify which gut bacteria deficiencies or overgrowths can lead to MAFLD, so as to specifically supplement or reduce these gut microbiota. Secondly, identifying the types of gut bacteria with therapeutic effects, as well as the metabolic products and components of gut bacteria linked to MAFLD, is crucial. Identifying the metabolic products and components of gut bacteria linked to MAFLD is essential. Consequently, additional research is required to confirm the long-term effects and safety of gut microbiota modulation therapy, as well as to develop more effective and safer treatment options for patients with MAFLD. **Table 4** outlines the major clinical and animal studies exploring the therapeutic effects of FMT on MAFLD. The summarized data highlight key shifts in gut microbiota alongside improvements in liver steatosis, inflammation, and overall metabolic health.

**Table 4:** Clinical and animal evidence for the therapeutic efficacy of faecal microbiota transplantation in MAFLD.

Study setting and model	FMT protocol (donor source + administration route)	Key microbiota changes after FMT	Main metabolic outcomes	BA/SCFA changes	Safety/tolerability	Ref.
Clinical studies						
Randomized controlled trial in MASLD patients (n=20)	Donor: two screened lean donors; route: gastroduodenoscopic infusion; comparator: autologous FMT.	Recipient *Akkermansia muciniphila* decreased after donor D08 FMT at weeks 3 and 12	Triglycerides showed a downward trend	—	—	(202)
Randomized controlled trial in biopsy-proven NAFLD patients (n=21)	Donor: vegan-diet healthy donors; route: oral capsules or nasoduodenal tube; comparator: autologous FMT.	Allogenic FMT increased Eubacterium siraeum and Blautia wexlerae and decreased Lactobacillus delbrueckii	Metabolic outcomes were not consistently reported	—	—	(203)
Animal studies						
HFD-induced NASH mouse model (3 groups, n=12/group)	Donor: fresh feces from control mice; route: oral gavage.	Increased Firmicutes and Actinobacteria; decreased Bacteroidetes; increased Lactobacillus and Prevotellaceae-related taxa; decreased Odoribacter and Oscillibacter	Reduced body weight, liver index, ALT/AST, hepatic TG/cholesterol and NAS score; improved inflammation, gut barrier and endotoxemia	Cecal butyrate increased, whereas acetate and propionate were unchanged	—	(156)
HFCS-induced MAFLD mouse model (n=5/group)	Donor: fresh feces from healthy control mice; route: oral gavage.	Higher alpha diversity, lower Firmicutes/Bacteroidetes ratio and increased Bifidobacterium	Lower body weight, ALT and TC; improved steatosis, lobular inflammation and NAS score	—	—	(204)
HFD-induced NAFLD mouse model (n=10/group)	Donor: single healthy human donor or pooled healthy human donors; route: oral gavage.	Decreased Proteobacteria; increased Lachnospiraceae_NK4A136_group, Akkermansia, Blautia and Bacteroidetes	Lower body weight, ALT, LDL-C, TC, TG and LPS; higher HDL-C; markedly attenuated steatosis	—	—	(201)
HHFCF-induced NASH mouse model	Donor: fresh feces from normal-diet mice; route: oral gavage.	Enriched Clostridium	Reduced liver weight, ALT/AST, steatosis, fibrosis markers and oxidative stress	—	—	(205)
HFD-induced NAFLD mouse model	Donor: mice pretreated with yellow-tea polysaccharides; route: oral gavage.	Decreased Streptococcus and Lactobacillus	Improved liver metabolic injury and lipid deposition; lower hepatic TG/TC and AST/ALT	Colonic DCA, HCA, HDCA, LCA and CA decreased	—	(206)
WD-induced MAFLD mouse model (n=6/group)	Donor: MAFLD patients before *vs* after HT intervention; route: oral gavage.	Increased Fusicatenibacter saccharivorans	Lower body and liver weight; improved OGTT/ITT and insulin sensitivity; lower hepatic TC/TG and serum LDL-C, ALT and AST; higher HDL-C	Faecal butanoic acid and acetic acid increased	—	(207)

### 6.5 Atherosclerosis

AS is a chronic illness that is strongly related to metabolic diseases. It is defined by the increasing accumulation of lipids in the artery walls as well as continuing inflammatory reactions. Recent research indicate that alterations in gut flora may significantly influence the onset and progression of AS (133, 208, 209). FMT has demonstrated potential in controlling gut microbiota in AS, according to preliminary study. FMT can affect metabolic and inflammatory responses by reinstating a healthy gut microbiota composition (210). In a study, faeces from wild-type mice were transplanted into CTRP9 gene knockout mice, resulting in a considerable enhancement of gut microbiota composition in the recipients, characterised by an increased proportion of Firmicutes and a decreased proportion of Bacteroidetes (211). This indicates that FMT may somewhat impede the advancement of atherosclerosis by re-establishing the equilibrium of gut bacteria. FMT may potentially mitigate atherosclerosis by reducing systemic inflammation. Zhao et al. found that by transplanting feces from healthy mice into Parkinson’s disease mice, the number of harmful bacteria was significantly reduced, and the abundance of beneficial bacteria in the gut was restored. After FMT treatment, the levels of endogenous inflammatory markers (TNF-α, IL-1β, and IL-6) were significantly reduced in the whole-body of the studied mice (212, 213), regulating T-cell differentiation and macrophage polarization (M1 to M2 transformation) (214, 215), which may slow the development of atherosclerosis by inhibiting inflammation in the arteries. Furthermore, after transplanting a pro-inflammatory microbiota (such as that from *Casp1−/−* mice) into low-density lipoprotein receptor-deficient (*Ldlr−/−*) mice, plaque area increased by 29%, confirming that the pathogenicity of the microbiota can be transmitted through FMT (214). In contrast, transplanting a healthy microbiota reversed the AS phenotype (211). Atherosclerosis is intimately linked to dyslipidaemia. FMT, by managing the gut microbiota and enhancing the lipid profile, may have a positive influence on the therapy of AS (216, 217).

Although the efficacy of FMT in AS is notable, current safety data are inadequate. FMT in AS treatment is generally safe, but more clinical observation and follow-up are required to ensure that adverse responses and potential dangers during the treatment process are identified and handled in a timely way. The current experimental evidence regarding the role of FMT in modulating gut microbiota to mitigate atherosclerotic lesions and systemic inflammation is summarized in **Table 5**. These preclinical models provide crucial insights into the mechanisms driving microbiota-mediated cardiovascular protection.

**Table 5:** Summary of experimental studies on faecal microbiota transplantation for the attenuation of atherosclerosis.

Study setting and model	FMT protocol (donor source + administration route)	Key microbiota changes after FMT	Main metabolic outcomes	BA/SCFA changes	Safety	Ref.
Animal studies						
FMT in CTRP9-knockout atherosclerotic mice (n=6/group)	Donor: WT *vs* CTRP9-KO mouse donors; route: oral gavage.	WT microbiota increased Firmicutes and Ruminococcaceae and decreased Bacteroidetes/Bacteroidales S24–7 group in KO recipients	Lower total cholesterol, LDL and TG; reduced carotid lesions, intimal hyperplasia, stenosis and aortic plaque area	—	—	(211)
CAP-induced ApoE-/- atherosclerosis model (n=10/group)	Donor: CAP *vs* control mice; route: oral gavage.	CAP-donor microbiota increased Firmicutes/Bacteroidetes ratio and Peptostreptococcaceae and decreased Odoribacteraceae	CAP microbiota aggravated atherosclerotic lesions	—	—	(218)
HFD-induced ApoE-/- atherosclerosis model (n=5/group)	Donor: ALW-II-41-27-treated *vs* vehicle-treated ApoE-/- donors; route: oral gavage.	Lower Firmicutes and higher Bacteroidetes; enrichment of Akkermansia, Enterococcus, Eggerthella and Lactobacillus	Reduced body weight, TC and LDL-C; smaller plaque area and necrotic core	Higher secondary/primary BA ratio, LCA and DCA	—	(219)
Exercise-donor transfer to HFD-fed atherosclerotic mice	Donor: aerobic-exercise donor mice; route: not reported.	Increased Lachnospiraceae NK4A136, Prevotellaceae_UCG-001 and Bacillus; decreased Faecalibaculum and Clostridium_sensu_stricto_1	Reduced body weight, lipid deposition, plaque burden, inflammation and LPS; improved dyslipidaemia	—	—	(220)
Validation study in western-diet-fed ApoE-/- mice (n=12/group)	Donor: aucubin-treated donor mice; route: oral gavage.	Increased Lactobacillus and Dubosiella	Lower serum TC, TG and LDL-C; higher HDL-C; reduced aortic and valvular plaque area	—	—	(221)
Validation study in CTRP9-deficient atherosclerotic mice	Donor: reciprocal WT and CTRP9-KO donors; route: oral gavage.	Therapeutic WT-to-KO FMT increased Firmicutes/Ruminococcaceae and decreased Bacteroidetes/S24-7, whereas KO-to-WT transfer showed the opposite pattern	WT-to-KO FMT lowered TC, LDL and carotid lesion area, whereas KO-to-WT transfer worsened these indices	—	—	(211)
Validation study in HFD-induced ApoE-/- mice (n=10/group)	Donor: HFD donor mice; route: oral gavage.	Increased Rikenella	Reduced plaque area and lipid deposition in the aortic root	—	—	(208)
Validation study in HFD-induced ApoE-/- mice	Donor: western-diet or pectin-supplemented donor mice; route: oral gavage.	Increased *Akkermansia muciniphila* and decreased Lactococcus lactis	Reduced aortic plaque area; lower TC, TG and LDL-C; higher HDL-C; attenuated hepatic lipid accumulation	Higher acetic acid and propionic acid	—	(222)
Validation study in HFD-induced ApoE-/- mice (n=6/group)	Donor: SGLT2i-treated donor mice; route: oral gavage.	Lower Firmicutes/Bacteroidetes ratio; higher Coriobacteriaceae, Lachnospiraceae, Ruminococcaceae and several SCFA-related genera	Reduced aortic plaque area	Lower CA, LCA, DCA and valerate	—	(209)

### 6.6 Advantages of FMT over other therapies

Compared with probiotics, prebiotics, and conventional pharmacological approaches, faecal microbiota transplantation (FMT) offer potential advantages in the management of GLMD. Although probiotics and synbiotics have shown some metabolic benefits in experimental and clinical settings, human evidence for sustained improvement in insulin resistance remains heterogeneous. For patients with complex disorders, supplementation with only one or a few strains may not be sufficient (223). Unlike therapies that mainly address a single downstream manifestation such as hyperglycaemia, dyslipidaemia, or obesity, FMT may exert broader systems-level effects across interconnected glucolipid metabolic pathways (224, 225). In individuals with hepatic steatosis or steatohepatitis, allogenic FMT altered gut microbiota composition and plasma metabolites and was associated with favourable changes in markers linked to hepatic inflammation and lipid metabolism, supporting the concept that FMT may influence multiple metabolic pathways rather than a single disease endpoint (226). FMT represents a “whole gut microbiome replacement” strategy and can transfer a more complete microbial ecosystem and functional network, potentially allowing a more profound restoration of microbial diversity and ecological balance (224, 227, 228). Compared with metformin, FMT may also have the advantage of broader ecological remodelling. Metformin partly acts through the gut microbiota, but current human data suggest that it mainly alters selected microbial taxa and does not produce consistent effects on overall microbial diversity. By contrast, FMT aims to achieve donor-microbe engraftment and ecosystem-level functional restoration (229, 230).

FMT serves as an adjuvant therapy rather than a replacement therapy. In a phase 2 trial, oral FMT combined with daily low-fermentable fibre supplementation improved insulin sensitivity in severe obesity with metabolic syndrome, providing proof of concept that microbiota transplantation can work synergistically with dietary therapy (162). Compared with bariatric surgery, FMT is less invasive and specifically microbiota-directed. Bariatric surgery has much stronger evidence for weight loss and diabetes remission, whereas FMT should at present be regarded as a microbiota-targeted adjunct or precision strategy rather than a replacement for metabolic surgery (231, 232). Current meta-analyses indicate that, although FMT may improve certain parameters such as insulin sensitivity or glycaemic index under specific circumstances, its overall efficacy remains heterogeneous and is still insufficient to support routine clinical application in metabolic diseases (158, 233). Thus, at present, the main advantage of FMT over other therapies lies more in its mechanistic breadth, ecological restoration capacity, and personalization potential than in definitively superior clinical outcomes. Future progress will depend on better donor screening, recipient stratification, optimization of engraftment, and standardized treatment protocols (224).

## 7 Heterogeneity perspectives of FMT

Human studies suggest that FMT can remodel the gut microbiome in GLMD, but its clinical benefits remain heterogeneous rather than uniform. In metabolic syndrome, lean-donor FMT transiently improved insulin sensitivity at 6 weeks, but this effect was not sustained at later follow-up, and treatment response was associated with lower baseline microbial diversity, supporting the existence of responder versus non-responder patterns (234). In obesity, several randomized trials showed sustained donor-related microbiome or bile acid shifts without significant reductions in BMI or consistent improvement in insulin sensitivity, indicating that microbiome engraftment does not necessarily translate into weight loss or broader metabolic benefit (158, 160, 235). Likewise, in adolescents with obesity, FMT did not significantly promote weight loss in the original randomized trial, although abdominal adiposity and *post hoc* metabolic syndrome resolution improved in some participants, and a 4-year follow-up later showed favourable changes in waist circumference, total body fat, metabolic syndrome severity, systemic inflammation, and HDL cholesterol despite no between-group difference in BMI (149, 161). This heterogeneity may arise from multiple sources. First, successful microbiota engraftment does not necessarily lead to immediate or clinically measurable metabolic improvement, because changes in microbial composition may be functionally insufficient or require longer time to affect host metabolism. Second, treatment response is likely influenced by differences in recipient baseline microbiota, metabolic status, diet, and inflammatory background, which may lead to distinct responder and non-responder patterns. In addition, variations in donor characteristics, transplantation protocols, dosing frequency, and concomitant interventions may further contribute to inconsistent clinical outcomes across studies.

The discrepancy between human and animal FMT studies should be interpreted as a translational gap rather than a contradiction. Animal models are valuable for establishing causality and dissecting mechanisms under highly controlled conditions, but they often simplify the clinical reality, because recipient genetics, diet, housing environment, microbiota depletion strategies, and disease phenotypes are much more uniform than in human populations (236–238). By contrast, clinical studies must contend with substantial inter-individual heterogeneity in baseline microbiota, metabolic status, medication exposure, diet, and immune background, all of which can influence donor-microbe engraftment, durability, and treatment response (224). Reducing this gap will require better alignment between preclinical and clinical research, including the wider use of humanized gnotobiotic models, more clinically relevant diets and phenotypes in animal experiments, standardized FMT protocols, and harmonized endpoints across trials (237, 238). Future FMT research should move beyond asking whether FMT works in general, toward identifying in whom, under which conditions, and through which microbial functions it can produce clinically meaningful and durable benefit.

## 8 Conclusion

FMT is increasingly recognised as a significant research focus in the treatment of GLMD. As the role of gut microbiota in the pathogenesis of GLMD is gradually revealed, FMT has shown its potential therapeutic effects in metabolic diseases such as diabetes, dyslipidaemia, and non-alcoholic fatty liver disease by restoring gut microbiota balance, alleviating gut barrier dysfunction, and reducing chronic low-grade inflammation. One important advantage of FMT over other therapies is its systems-level mechanism of action. Rather than targeting only a single clinical endpoint, FMT may simultaneously remodel gut microbial composition, restore microbial metabolites, strengthen the intestinal barrier, and reduce chronic inflammation, thereby addressing several interconnected drivers of GLMD at the same time. This may be particularly relevant in patients with multiple overlapping metabolic disorders. Nevertheless, FMT should currently be regarded as a promising adjunctive strategy rather than a replacement for established therapies, because further evidence is still needed regarding standardisation, long-term efficacy, and safety.

Despite the promising therapeutic prospects of FMT, its clinical application still faces many challenges, including precise selection of indications, donor screening and safety assessment, as well as long-term efficacy verification. Currently, FMT mainly relies on faecal transplantation from healthy donors, with its effects varying due to individual differences. Consequently, next research needs to focus on methodically screening particular probiotic populations and refining FMT implementation methodologies to attain personalised and exact treatment. Moreover, the mechanisms of FMT in treating GLMD require further investigation, particularly the specific pathways through which it influences the gut-liver axis, immune modulation, and metabolic remodelling.

## Glossary

GLMD: Glucolipid Metabolic Disorders
FMT: Faecal Microbiota Transplantation
TJs: Tight Junction
JAMs: Junctional Adhesion Molecules
IECs: Intestinal Epithelial Cells
TMA: Trimethylamine
PC: p-cresol
H2S: Hydrogen Sulphide
IgA: immunoglobulin A
NF-κB: Nuclear Factor-kappa B
LPS: Lipopolysaccharide
TLR4: Toll-like receptor 4
NF-κB: Nuclear Factor kappa B
TNF-α: Tumour Necrosis Factor-α
IL-6: Interleukin- 6
IL-1β: Interleukin- 1β
SCFAs: Short-Chain Fatty Acids
FXR: Farnesoid X Receptor
TMAO: Trimethylamine N-oxide
AS: Atherosclerosis
MAFLD: Metabolic-associated Fatty Liver Disease
MetS: Metabolic Syndrome
T2DM: Type 2 Diabetes Mellitus
HOMA-IR: Homeostatic Model Assessment of Insulin Resistance
BAs: Bile Acids
GPCRs: G Protein-Coupled Receptors
ROS: Reactive Oxygen Species
SIgA: Secretory Immunoglobulin A
ZO-1: Zonula Occludens-1
IL-22: Interleukin 22
IL-23: Interleukin 23
ILC3: Group 3 Innate Lymphoid Cells
BSEP: Bile Salt Export Pump
HDAC: Histone Deacetylase
LDL: Low-Density Lipoprotein
PAMPs: pathogen-associated molecular patterns
Ldlr−/−: Low-Density Lipoprotein Receptor Deficient.
